# Pd-Based Hybrid Nanoparticles As Multimodal Theranostic Nanomedicine

**DOI:** 10.1021/acsabm.2c00759

**Published:** 2023-01-18

**Authors:** Alberto Bellissima, Lorena M. Cucci, Vanessa Sanfilippo, Angela De Bonis, Roberto Fiorenza, Salvatore Scirè, Tiziano Marzo, Mirko Severi, Diego La Mendola, Valentina Notarstefano, Elisabetta Giorgini, Cristina Satriano

**Affiliations:** † Nano Hybrid BioInterfaces Laboratory (NHBIL), Department of Chemical Sciences, University of Catania, viale Andrea Doria, 6, 95125Catania, Italy; ‡ Department of Science, University of Basilicata, viale dell’Ateneo Lucano, 10, 85100Potenza, Italy; § Department of Chemical Sciences, University of Catania, viale Andrea Doria, 6, 95125Catania, Italy; ∥ Department of Pharmacy, University of Pisa, via Bonanno Pisano 6, 56126Pisa, Italy; ⊥ Department of Chemistry ‘‘U. Schiff’’, University of Florence, via della Lastruccia 3, 50019Sesto Fiorentino, Italy; # Department of Life and Environmental Sciences, Polytechnic University of Marche, Via Brecce Bianche, 60131Ancona, Italy

**Keywords:** plasmonics, photocatalytic activity, multimodal platform, prostate cancer cell targeting

## Abstract

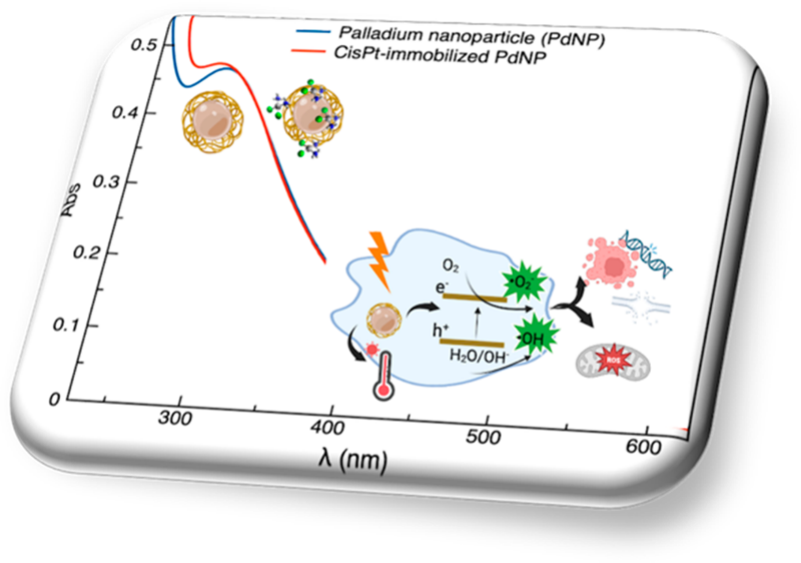

A nanodelivery system based on palladium nanoparticles (PdNP) and cisplatin (CisPt) was developed by physisorption of the drug onto the PdNP synthesized via a green redox process, using d-glucose and polyvinylpyrrolidone (PVP) as reducing and stabilizing/capping agents, respectively. UV–vis analysis and H_2_-evolution measurements were carried out to prove the nanoparticles’ capability to act as bimodal theranostic nanomedicine, i.e., having both plasmonic and photocatalytic properties. XPS, XRD, and TEM allowed light to be shed on the chemical composition and morphology of the PdNP. The analysis of the UV–visible spectra evidenced plasmonic peak changes for the hybrid nanoparticle-drug assembly (Pd@CisPt), which pointed to a significant interaction of CisPt with the NP surface. The drug loading was quantitatively estimated by ICP-OES measurements, while DLS and AFM confirmed the strong association of the drug with the nanoparticle surface. The test of SOD-like activity in a cell-free environment proved the maintenance of the antioxidant capability of PdNP also in the Pd@CisPt systems. Finally, Pd@CisPt tested in prostate cancer cells (PC-3 line) unveiled the antitumoral action of the developed nanomedicine, related to reactive oxygen species (ROS) generation, with a condition of protein misfolding/unfolding and DNA damage, as evidenced by cytotoxicity and MitoSOX assays, as well as Raman microspectroscopy, respectively. Cell imaging by confocal microscopy evidenced cellular uptake of the nanoparticles, as well as dynamic processes of copper ion accumulation at the level of subcellular compartments. Finally, cell migration studies upon treatment with Pd@CisPt evidenced a tunable response between the inhibitory effect of CisPt and the enhanced rate of cell migration for the metal NP alone, which pointed out the promising potential of the developed theranostic nanomedicine in tissue regeneration.

## 1 Introduction

Nanomedicine and nanoparticle (NP)-based drug delivery have emerged as innovative therapeutic approaches for preventing drug-related toxicity and overcoming drug resistance, a major impediment to cancer treatment.^1^

NPs can accumulate at the tumor site, due to the enhanced permeability and retention (EPR) effect, which is related to the leaky vasculature and poor lymphatic drainage in the tumor microenvironment. For a successful treatment and simultaneous diagnosis (so-called theranostics) of cancer, a very promising strategy is the development of multifunctional nanocarriers incorporating both cancer diagnostics and therapeutic capabilities.^2^

Cisplatin [*cis*-diamine platinum(II) dichloride] (CisPt), a widely used chemotherapeutic, is the first FDA-approved platinum-based drug.^3^ The cytotoxicity of CisPt is known to be mediated by its interaction with DNA, resulting primarily in intrastrand-cross-linked DNA adducts, which in turn activate signal transduction pathways culminating in the apoptosis activation.^4^

The cisplatin-based therapy, successful in various types of solid tumors, has however several drawbacks, including severe side effects and drug resistance, caused by the inefficient or insufficient delivery to cancer cells of the cytotoxic drug.

Mitochondria are potential therapeutic targets for several anticancer drugs, including CisPt. In fact, CisPt tends to accumulate in mitochondria, damaging their structure and metabolic function.^5^ In particular, the cell sensitivity to cisplatin-induced apoptosis is critically determined by the mitochondrial content and the related capacity of mitochondria to produce ROS.^6^

In recent years, Pd nanoparticles (PdNP), owing to their excellent biocompatibility and high stability in the physiological environment, have shown a very high potential for nanomedicine. Like other noble metals, Pd is getting great attention in biomedical applications for its plasmonic features, resulting in remarkable electronic and optical properties; moreover, compared with the most intensively studied gold and silver nanoparticles,^7^ Pd-based nanomaterials exhibit distinctive features, including high photothermal conversion efficiency, high chemical stability, higher susceptibility of the localized surface plasmon resonance peak to environmental refractive index changes,^8^ and substantial photocatalytic activity.^9^ A broad use of Pd nanomaterial is as catalysts, related to its high affinity for hydrogen.^10^ Pd nanoparticles have wide applications such as glucose sensors and fuel cells.^11^

Pd-based nanomaterials are also highly versatile catalysts suitable in organic synthesis (catalyst in C–C coupling reactions and biocatalyst) and ROS inducing cancer treatment reagents. As an example, Pd nanocubes have been suggested as apoptosis inducing agents, due to their ability to generate singlet oxygen species simply by chemisorption of molecular oxygen on the NP surface and without photoexcitation.^12^

The chemo-photothermal therapy synergistic effect of Pd-based nanomaterials has been exploited with peptide-functionalized PdNPs as a dual stimuli-responsive drug delivery system^13^ and in nano Pd-decorated manganese dioxide nanosheets for controlled drug release in the tumor site by photothermal effect under a near-infrared (NIR) stimulus.^14^

In this work, we assembled a multimodal nanoplatform that combines the plasmonic and the photocatalytic properties of Pd nanoparticles with the anticancer activity of CisPt. By this “catalytic metallodrugs” approach,^15^ we exploited the compatibility of the transition-metal-based theranostic system in a biological environment, namely, an aqueous, oxygen-containing medium, as well as its capability of performing bioimaging and transformations inside cells.

## 2 Experimental section

### 2.1 Chemicals

Anhydrous palladium chloride was purchased from Thermo Fisher Scientific. d-(+)-Glucose, polyvinylpyrrolidone (PVP; average molecular weight: 44 000 Da), sodium hydroxide (NaOH, 97%, pellet), and hydrochloric acid (HCl, 37% in H_2_O) were obtained from Sigma-Aldrich. A phosphate buffer saline solution (PBS; 0.01 M phosphate buffer, containing 0.003 M KCl and 0.14 M NaCl, pH 7.4) was prepared from tablets (Sigma-Aldrich). For all experiments, ultrapure water (18.2 MΩ·cm at 25 °C, total organic carbon (TOC) less than 5 parts per billion (ppb), Millipore Ultrapure Water Type) was used. Glassware was cleaned with aqua regia solution (HCl/HNO_3_ 3:1 volume ratio) and rinsed with ultrapure water prior to each use. For the SOD-like activity assay in a cell-free environment, xanthine, xanthine oxidase, and cytochrome c were purchased from Sigma-Aldrich. For the cellular experiments, RPMI-1640 medium, penicillin-streptomycin, l-glutamine, fetal bovine serum (FBS), dimethyl sulfoxide (DMSO), Dulbecco’s PBS, paraformaldehyde, and 3-(4,5-dimethylthiazol-2-yl)-2,5-diphenyltetrazolium bromide (MTT) were purchased from Sigma-Aldrich. A ReadyProbes Cell Viability Imaging Kit Blue/Green (Hoechst 33342 NucBlueTM Live reagent and NucGreenTM Dead cell reagent) and MitoSOX dye were purchased from Thermo Fisher Scientific. TiO_2_ P25 Aeroxide (80% anatase, 20% rutile, 50 m^2^/g surface area, < 100 nm of particle size) was used as purchased from Acros Organics.

### 2.2 Preparation of PdNP

The palladium nanospheres were synthesized using a green reduction method, modifying the procedure reported by Xu et al.^16^d-(+)-Glucose was used to reduce Pd^2+^ to Pd^0^, with the addition of NaOH to neutralize it and PVP as a stabilizing agent. Briefly, a 60 mM palladium salt solution was obtained by dissolving PdCl_2_ (10.6 mg/mL) in 2 mL of HCl (0.2 M) in a glass vial, at 60 °C under stirring, until a bright orange solution was obtained. Then, PVP (0.2 mM) was added to the solution as a stabilizing reagent. To reduce the palladium salt, the prepared mixture was added to 10 mL of 0.6 M d-(+)-glucose aqueous solution, in a 20 mL glass vial at room temperature, with moderate stirring. Finally, NaOH (1.0 M) was added dropwise to the reaction mixture until a pH of 7.4 was reached, which produced a color change from orange to brown, due to the formation of PdNP colloids. According to the literature,^16^ the measured plasmonic peak at 274 nm pointed to the formation of Pd nanoparticles of about 5 nm in diameter. For purification, the nanoparticle dispersions were centrifuged twice (1 min, 8000 r.p.m) using an Amicon tube (30 kDa cutoff filter), with a washing with 10 mM PBS buffer (pH = 7.4) in between the two centrifugation steps, to remove the PdCl_2_ and glucose excess and to concentrate the nanosystems.

### 2.3 Preparation of Pd@CisPt Hybrids

The PdNP pellet, obtained as described in section 2.2, was resuspended in 10 mM PBS at a concentration of 158 nM (3.3 × 10^11^ NP/ml) and then functionalized with the drug by physisorption of CisPt from a 2.4 mM solution. Such a concentration corresponds to the limit value before the aggregation occurred when CisPt was added to the nanoparticle dispersion. Unbounded or weakly bound CisPt molecules were rinsed off by two steps of centrifugation (1 min, 8000 r.p.m.) in 10 mM PBS by using an Amicon tube (30 kDa cutoff filter), with buffer rinsing in between the two centrifugation steps.

### 2.4 Physicochemical Characterization

#### 2.4.1 UV–Visible (UV–vis) Spectroscopy and Dynamic Light Scattering (DLS)

UV–vis spectra were collected in the wavelength range of 200–700 nm with a PerkinElmer Lambda 365 UV–vis spectrometer at 25 °C, with quartz cuvettes (0.1 cm optical path), with a spectral resolution of 1 nm. The particle hydrodynamic size analysis was performed using a DLS (Horiba LB-550) instrument. Results are presented as means ± standard deviation (SD) of at least three measurements.

#### 2.4.2 Photocatalytic Activity

Photocatalytic H_2_ production was investigated to determine the photocatalytic performance of PdNP. The tests were carried out using a Pyrex jacketed batch reactor irradiated with a solar lamp (OSRAM Vitalux 300 W, 10.7 mW/cm^2^). Twenty-five milligrams of sample were mixed with 50 mL of a water–glycerol (employed as a scavenger) solution (40 and 10 mL, respectively) under constant stirring, keeping the temperature at 35 °C. The mixture was purged 1 h with argon to eliminate dissolved air. The suspension was then irradiated for 5 h. The evolved H_2_ was quantified with an Agilent 6890 gas chromatograph (Carboxen 1000 packed column, TCD detector, and Ar as gas carrier).

#### 2.4.3 X-ray Photoelectron Spectroscopy (XPS)

XPS measurements were performed using a SPECS Phoibos 100- MCD5 spectrometer, using Mg Kα (1253.6 eV) radiation. Spectra were acquired with a channel width of 0.1 eV and analyzed by Googly, a curve-fitting program that allows the simultaneous fitting of peaks in the form of Voigt functions and their associated background in a wide energy range.^17^ Peak positions were referenced to the C 1s aliphatic carbon set at 285.0 eV.

#### 2.4.4 Transmission Electron Microscopy (TEM)

The Pd colloidal solution was drop casted onto a copper grid of holey carbon films (400 mesh, AGAR Scientific) for TEM characterization (FEI-TECNAI, 200 kV). More than 600 NPs were measured to obtain the nanoparticle size histograms by ImageJ software.

#### 2.4.5 X-ray Diffraction Spectroscopy (XRD)

For XRD analysis, PdNP colloidal solution was drop casted onto a monocrystalline (100) silicon substrate. Diffraction spectra were collected with the Bruker D5000 X-ray diffractometer (Cu Kα radiation, 40 kV and 32 mA) at 2θ angles of 30–50 and a step size of 0.04.

#### 2.4.6 Atomic Force Microscopy (AFM)

AFM topographic images were collected using a Cypher S instrument (Asylum Research, Oxford Instruments) operating in tapping mode. During the operations, silicon tetrahedral tips were used, mounted on rectangular 30 μm cantilevers, purchased from Olympus (AC240TS, Oxford Instruments). The probes had a nominal spring constant of 2 N/m and driving frequencies of 70 kHz. To perform the analysis, samples were deposited on freshly exfoliated muscovite mica (Ted Pella, Inc.). After 5 min from the moment of deposition, mica dishes were washed with 1 mL of ultrapure water and dried under a gentle nitrogen flow.

#### 2.4.7 Inductively Coupled Plasma Optical Emission Spectrometry (ICP-OES)

Pt and Pd concentrations were determined using a Varian 720-ES Inductively Coupled Plasma Atomic Emission Spectrometer (ICP-AES) equipped with a CETAC U5000 AT+ ultrasonic nebulizer, to increase the method sensitivity. Before the analysis, each sample was spiked with 100 μL of a 50 mg L^–1^ Ge solution, used as an internal standard, and then analyzed. Calibration standards were prepared by serial gravimetric dilution of a commercial standard solution of Pt and Pd at 1000 mg L^–1^. The wavelength used for the metal determination were 214.424 nm for Pt, 360.955 nm for Pd, and 209.426 nm for Ge, respectively. The operating conditions were optimized to obtain maximum signal intensity, and between each sample a rinse solution of aqua regia (2% v/v) was used to avoid any “memory effect”.

### 2.5 Biochemical and Biological Characterization

#### 2.5.1 Superoxide Dismutase (SOD)-Like Activity

The SOD-like activity of the free cisplatin, the bare, and the CisPt-loaded PdNP was determined by the indirect Fridovich method.^18^ Xanthine and xanthine oxidase were used to generate superoxide anion radicals, which were detected spectrophotometrically by monitoring the reduction of cytochrome c, which shows an absorbance band at 550 nm.

The measure was carried out by mixing in 10 mM pH 7.4 phosphate buffer the different samples with 50 μM xanthine, 10 μM cytochrome c, and the appropriate amount of xanthine oxidase to produce a Δ*A*/min of 0.025. UV–vis spectra (number of cycles = 5, cycle time = 1 min) were recorded on a PerkinElmer Lambda 365 UV–vis spectrometer using 1 cm quartz cuvettes.

#### 2.5.2 Cell Culture and Treatments

Prostate cancer cells (PC-3 line) were cultured in 25 cm^2^ plastic flasks using complete growth medium (i.e., RPMI-1640 medium supplemented with 2 mM l-glutamine, 100 UI penicillin, 0.1 mg/mL streptomycin, and 10% v/v FBS) in a Heraeus Hera Cell 150C incubator, under a humidified atmosphere at 37 °C in 5% CO_2_.

One day before the cellular experiments, cells were seeded in 96-well plates (10^4^ cells/well; cytotoxicity and mitochondrial ROS assays) or in glass bottom dishes with a 12 mm glass diameter (WillCo-dish, Willco Wells, B.V.; 2 × 10^4^ cells/plate; confocal microscopy and Raman microspectroscopy) or in 48-well culture plates (1.8 × 10^5^ cells/well; cell migration assay) in FBS (10% v/v)-supplemented RPMI 1640 medium.

Just before the treatments, cells were rinsed with RPMI-1640 medium supplemented with 2 mM l-glutamine, 100 UI penicillin, 0.1 mg/mL streptomycin, and 1% v/v FBS, then incubated at 37 °C in a 5% CO_2_ atmosphere with the different samples at the specific times/concentrations chosen for the cellular experiment (see below). In all experiments, the negative control of untreated cells (Ctrl) was included.

#### 2.5.3 Cytotoxicity and Mitochondrial ROS Production Assays

Cells were incubated for 24 h at the following treatment conditions: CisPt (1.8–18 μM), PdNP (2–20 nM), and Pd@CisPt (NP/drug concentration = 2 nM/1.8 μM, 10 nM/9 μM, 20 nM/18 μM).

The cytotoxicity of the samples was tested in PC-3 cells using two colorimetric tests to probe, as an indicator of cell viability, nuclear protein/nuclear acid staining (ReadyProbes Cell Viability Imaging Kit, Invitrogen) and reduction of the yellow MTT tetrazolium salt to purple formazan crystals by metabolically active cells (MTT assay), respectively.

Nuclear staining of total cells or only dead cells was carried out by incubation at 37 °C for 30 min of cells, respectively, with NucBlue Live reagent (excitation/emission: 360/460 nm) and NucGreen Dead reagent (excitation/emission: 504/528 nm). Afterward, the solutions from triplicate dishes were collected by mechanical scraping of the wells and unified to record the fluorescence emission spectra on a PerkinElmer LS55 fluorimeter. For data analysis, the ratio between the NucBlue and NucGreen signals was reported as the percentage of viable cells compared to the untreated cells.

For the MTT assay, cytotoxicity was determined by incubating cells at 37 °C with the MTT solution (5 mg/mL), then detecting the enzymatic reduction of MTT in the insoluble purple formazan product by dissolving the crystals with 100 μL of DMSO and measuring the absorbance at 570 nm on a Varioscan spectrophotometer. All experiments were carried out in triplicate, and the results are presented as means ± SD. The statistical analysis was performed by Student’s *t* test.

The oxidative stress was evaluated through the MitoSOX assay by measuring the level of ROS (specifically, mitochondrial O_2_•−) after 24 h of PC-3 cell treatment with the different samples. To stain cells, a 20 min treatment with Hoechst33342 (0.12 μg/mL) was performed, followed by 5 min of incubation at 37 °C with MitoSOX (5 μM). Samples were analyzed by measuring fluorescence emission (ex/em = 358/461 nm for nuclear staining; ex/em = 510/580 nm for MitoSOX, respectively) using an LS55 fluorescence spectrophotometer (PerkinElmer) and quartz cuvettes with an optical path length of 0.1 cm. Results, normalized to the Hoechst emission and represented as the increase in MitoSOX signals with respect to the untreated control, are presented as the means ± SD of three replicas.

#### 2.5.4 Laser Scanning Confocal Microscopy (LSM)

LSM analyses were carried out with an Olympus FV1000 confocal laser scanning microscope, equipped with diode UV (405 nm), multiline argon (457 nm, 488 nm, 515 nm), and HeNe(G)/(R) (543/633 nm) lasers. The detector gain was fixed at a constant value. Spectral filtering systems were used, and images were collected by using an oil immersion objective (60xO PLAPO), in sequential mode, randomly throughout the area of the well. The image analysis was carried out using Huygens Essential software (by Scientific Volume Imaging B.V.). The statistical analysis was performed using Student’s *t* test.

After 24 h of treatment with CisPt (18 μM), PdNP (17 nM), and Pd@CisPt (NP/drug concentration: 20 nM/18 μM, sample named Pd@CisPt1000), cells were stained with the nuclear dye Hoechst33342 (1 μg/mL) and MitoTracker deep red (2 × 10^–7^ M) and the intracellular copper probe (1 × 10^–6^ M) before performing live confocal imaging. Finally, cells were fixed with high purity paraformaldehyde (2% in PBS, pH = 7.3).

#### 2.5.6 Raman Microspectroscopy (RMS) Measurements and Data Analysis

Cells were incubated for 24 h at the following treatment conditions: CisPt (18 μM), PdNP (20 nM), and Pd@CisPt hybrids (NP/drug concentration: 20 nM/18 μM, sample named Pd@CisPt1000; 20 nM/9 μM, sample named Pd@CisPt500).

A Horiba Jobin-Yvon XploRA Raman microspectrometer with a light source from a 532 nm diode laser (∼50 mW laser power at the sample) and a 100× objective (Olympus) was used. Spectra were acquired for 3 × 10 s at each spot in the 600 to 1800 cm^–1^ spectral range. Before starting acquisitions, the calibration of the spectrometer was performed with reference to the 520.7 cm^–1^ line of silicon. A grating with 1800 lines per mm (∼1 μm spot size and a 100 μm confocal pinhole were used for all measurements); spectra were dispersed onto a 16-bit dynamic range Peltier-cooled CCD detector.

In each cellular sample, Raman maps on ∼40 single cells were acquired with a spatial resolution of 2 μm. From each map, the spectra of the nuclear region were extracted and averaged (averaging routine, OPUS 7.5 software, Bruker Optics GmbH). The contribution of glass to the spectra was removed by the extended multivariate signal correction algorithm (EMSC). Pairwise PCA was performed on the entire spectral range of preprocessed spectra of Ctrl/CisPt, Ctrl/PdNP, and Ctrl/Pd@CisPt. PC scores and loadings were considered (OriginPro 2018b, OriginLab Corporation).^19^ The height (*I*) of some selected peaks with biological meaning was calculated (Integration mode K, OPUS 7.5 software) to quantify the alterations due to treatments.

#### 2.5.7 Wound Healing Assay

At cell confluence, the scratching of cell monolayers was performed by using a universal sterile 10-μL pipet tip; followed by cell rinsing with FBS (1% v/v)-supplemented RPMI 1640 medium. Immediately after the scratch, plates were marked to ensure that the serial phase-contrast images (Leica) of *in vitro* wounds were taken immediately after treatment (*t* = 0) and after 3, 5, 24, and 48 h of incubation precisely at the same location throughout each experiment.

The treatments conditions were as follows: CisPt, 18 μM; PdNP, 20 nM; Pd@CisPt, 20 nM/18 μM NP/drug concentration. The separation wall width was measured by using the ImageJ software, with the MRI Wound Healing Tool macro (version 1.50i, NIH).

## 3 Results and Discussion

### 3.1 Physicochemical Characterization of PdNP and Pd@CisPt Nanomaterials

**Figure 1:**
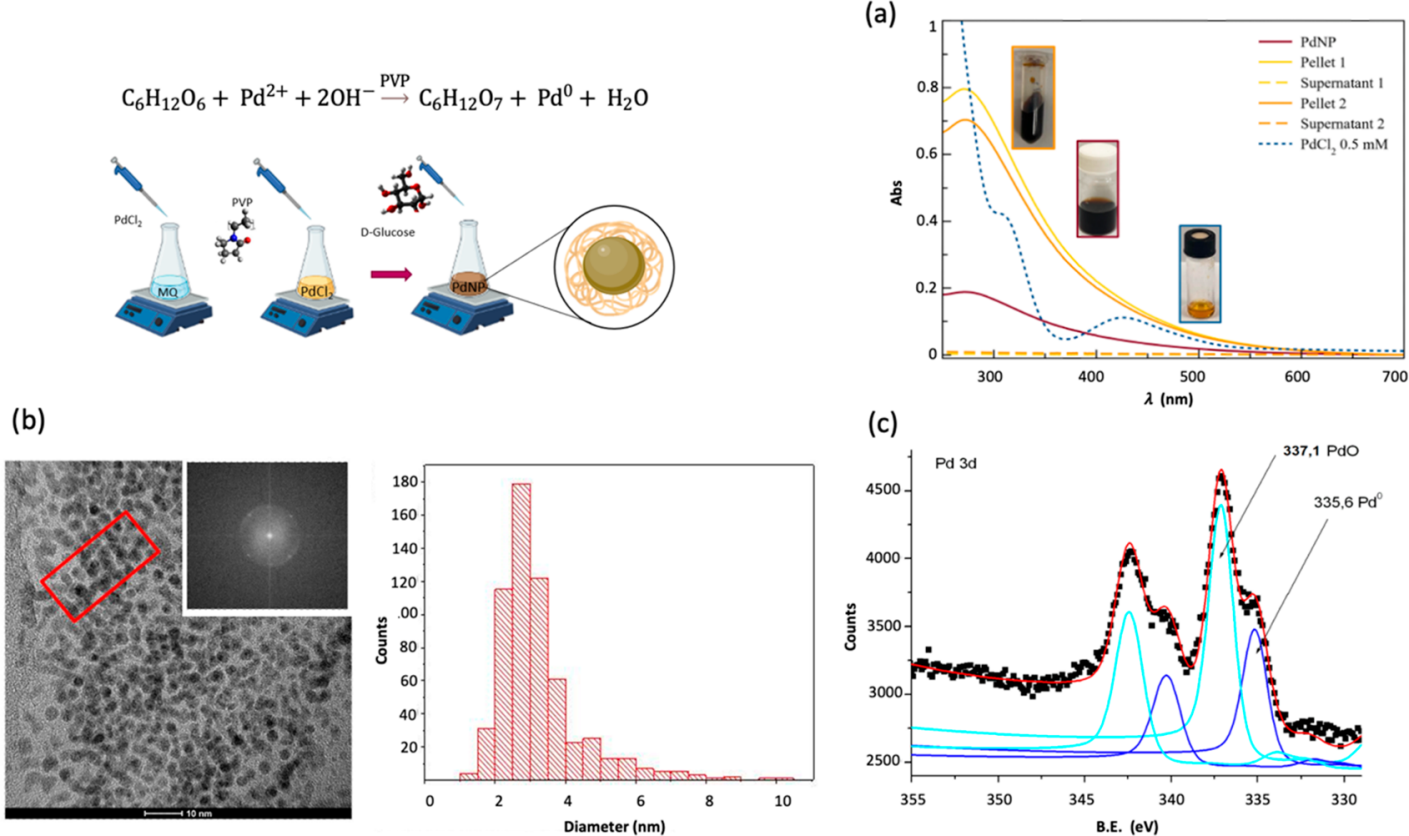
(a) UV–visible spectra of PdNP (wine solid line) before and after the two centrifugation steps in 10 mM PBS at room temperature (1 min, 8000 rpm) for the resuspended pellets 1 (yellow, solid line) and 2 (orange, solid line), respectively; the supernatants spectra (dashed lines) collected after the two centrifugation steps, as well as the reference spectrum of palladium chloride (blue, dotted line) are also included. (b) TEM image of PVP-PdNP, size distribution (>600 particles have been measured), and 2D-FFT image of the area in the red box. (c) XPS spectrum of Pd 3d core-level doublets for PVP-stabilized PdNPs. Pd is present in two oxidation forms: Pd_3/2_ and Pd_5/2_ doublet of Pd^0^ (blue lines) and Pd_3/2_ and Pd_5/2_ doublet of Pd^2+^ (cyan lines).

A green synthesis of PdNP using d-(+)-glucose as the reducing agent^16^ was implemented with the addition of poly(vinylpyrrolidone) (PVP) as the stabilizing agent (Figure 1).

The reaction evolution, monitored by UV–vis spectroscopy, exhibits a gradual change of the color from orange (due to ligand-to-metal charge transfer transitions (LMCT) of the Pd^2+^ complex in hydrochloric acid solution; Figure 1a, blue line) to dark brown (due to the excitation of the surface plasmon resonance of Pd nanoparticles; Figure 1a, wine line).^20^ After a reaction time of a few minutes, the reduction of Pd^2+^ ions to the metal Pd^0^ is complete, and the peak at 420 nm disappears while the plasmonic peak of PdNP at around 273 nm is observed in the spectrum.

The UV–vis spectra of the nanosystems after the rinsing steps in PBS and the following purification/concentration procedure (Figure 1a, yellow and orange lines), show the maintenance of the distinctive plasmon peak at around 273–274 nm, suggesting that no aggregation effect occurs during the centrifugation steps. However, a slight decrease of the maximum absorbance is more evident for pellet 2 than for pellet 1, to some extent due to the loss of particles in the Amicon tube filter during the washing step.

From the optical spectra of the PdNPs, the average core diameter size (*d*) and extinction coefficient (ε) were estimated, according to the following equations:
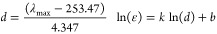
where λ_max_ is the wavelength at the absorption peak,^16^ and *k* (=2.88) and *b* (=10.48) are constants.^21^

The calculated optical size and the molar concentration of the PdNP dispersion by using the Lambert–Beer law of solutions are given in the Table S1.

The TEM micrograph confirms the formation of spherical NPs (Figure 1b). In quite good agreement with the optical diameter calculated from the optical spectra, the histogram distribution of the particle diameter shows that the particles have a median diameter of 2.9 nm with a standard deviation of σ_d_ = 1.3 nm. Moreover, high-resolution TEM images indicate the crystalline domains of single particles. The plane spacing of the observed crystalline domains is 0.22 nm, as evaluated by the respective 2D-fast Fourier transformation (2D-FFT) patterns (Figure 1b). The measured spacing is consistent with the lattice distance of the planes (111) of the FCC crystal structure of metallic Pd.^22^

The chemical valence of the PdNP system was studied by XPS analysis. The deconvoluted spectrum of the Pd 3d core level is composed of two Pd_3/2_ and Pd_5/2_ spin–orbit doublets (Figure 1c). The peaks centered at a B.E. of 335.6 and 337.1 eV are assigned to the Pd 3d_5/2_ component of metallic (Pd^0^) and divalent (PdO) forms, respectively. The data obtained corroborate those available from the literature.^23^ Indeed, in the nanoparticle, Pd exists in two different oxidation states where the main component is the divalent form that accounts for about 65% of the Pd.

The XRD analysis of the nanoparticles deposited onto a monocrystalline silicon substrate confirms that palladium is present in the two different forms of cubic PdO, with (200) and (220) crystal planes, and cubic Pd, with (111) crystal planes (Figure S1)

To test the catalytic activity of the Pd nanoparticles, they were impregnated (by the wetness impregnation method) on the TiO_2_ P25 support to obtain a palladium weight percentage of 0.1. After impregnation, the sample was dried at 70 °C overnight. The results analysis (data illustrated in the Figure S2) indicated that the addition of the Pd nanoparticles boosts the solar H_2_ production. The Pd/TiO_2_ sample showed, in fact, a H_2_ evolution about 19 times higher than that of bare TiO_2_, which is the most used photocatalyst for this reaction.^24^ In particular, the H_2_ evolution was tested by solar photocatalysis only to verify the photoresponsive properties of the as-prepared NPs and not to directly test their anti-inflammatory effects. However, in the current contest, the high photocatalytic activity of our PVP-coated PdNP, which significantly improved solar H_2_ production despite the low amount of Pd (0.1 wt %) loaded on TiO_2_, represents a very promising proof-of-work result for the application of the developed nanomaterials as a nanozyme-theranostic platform. The H_2_ production obtained after 5 h of artificial solar irradiation (383.5 μmol/g_cat_.·h) was, in fact, higher or comparable to other Pd/TiO_2_-based photocatalysts reported in the literature (Table S2).

**Figure 2:**
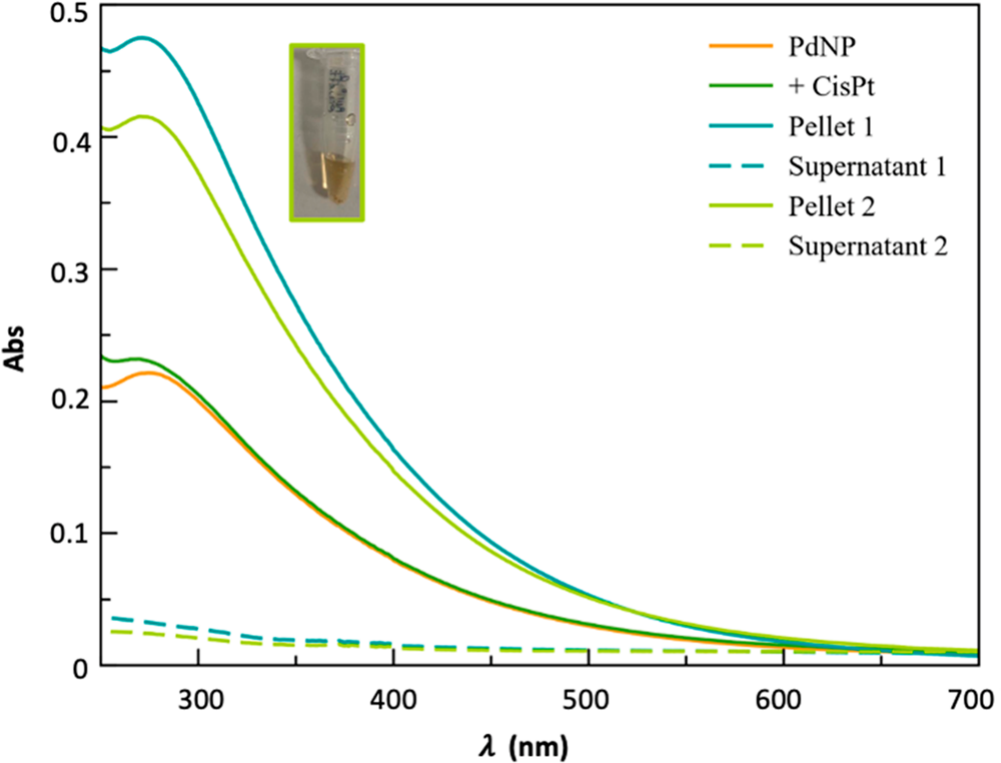
UV–visible spectra of PdNP (1.58 × 10^–7^ mol/L, 1.8 × 10^12^ NP/mL; orange solid line) before and after the addition of 2.4 mM CisPt (green solid line). The dark-green and light-green solid curves refer to the first and second pellets, respectively, while the dashed lines refer to the supernatants, obtained after two centrifugation steps in 10 mM PBS at room temperature (1 min, 8000 rpm). All spectra were recorded by diluting the mother solution five times in 10 mM PBS and using a quartz cuvette (optical path length = 0.1 cm).

In Figure 2, we show the optical spectra of PdNP both before and after functionalization with CisPt. It is known that a variation in the refractive index of the medium surrounding a plasmonic nanoparticle causes shifts in its plasmon extinction band.^25^ This variation was extensively utilized to detect analytes as well as to demonstrate the effective interaction of drug molecules with the nanoparticle surface. Indeed, the addition of CisPt to the PdNP caused a hypsochromic shift of the plasmon peak (Δλ_max_ = 9 nm; green line in Figure 2). It should be noted that the UV–vis spectra of the hybrid Pd@CisPt after the purification procedure show that the distinctive plasmon peak shift is enhanced (cyan and light green lines in Figure 2).

Regarding purification, Amicon filter units with a 3 or 30 kDa cutoff point for the centrifugation steps were compared. Using the 30 kDa cutoff, a higher concentration of nanoparticles was collected, due to a minor filling of the filter, which leads to less aggregation. This was especially evident for the Pd@CisPt pellet sample, which resulted in an 8-fold higher concentration relative to the centrifuged system with a 3 kDa cutoff, as estimated considering the absorbance value at 550 nm. This value has been demonstrated to be optimal to achieving high sensitivity and minimizing the error for the experimental analysis. In fact, the UV–vis spectrum of PdNP suffers from the increase of the signal-to-noise ratio as the wavelength decreases and becomes considerably steeper at lower wavelengths (e.g., 200–400 nm), with a small deviation in the wavelength resulting in a very large error in the absorbance. According to the above, the wavelength near 550 nm represents the ideal balance between these two factors, which is advantageous for the theoretical calculations (see discussion in SI).

Notably, the procedure used for the loading of PdNP proved to be highly reproducible. In fact, cisplatin loading was independently measured through ICP-OES analysis (see Experimental Section for details). The resulting concentration of CisPt (9.07 × 10^–4^ M) was superimposable with that independently calculated (Table S3). Additionally, this multielemental technique allowed us to measure the Pt/Pd molar ratio, which has been found to be 0.26.

The Pd-based nanomaterials, before and after functionalization with cisplatin, were investigated by DLS.^26^ The values showed an increase in the average hydrodynamic size from the prepared PdNP (27 ± 4 nm) to the purified pellet (45 ± 3 nm, effect of removal of excess reactant and partial aggregation^27^) used as a substrate for drug physisorption then another increase (75 ± 4 nm) in Pd@CisPt samples.

The AFM analyses confirmed the spherical shape of Pd nanoparticles and their effective functionalization with cisplatin.

**Figure 3:**
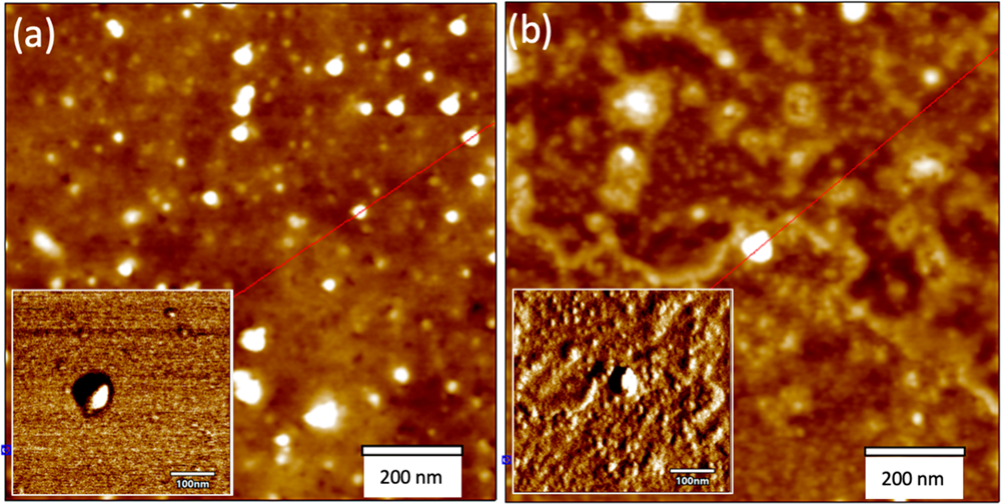
AFM images recorded in AC mode in air of height (*z* scale = 10 nm) and phase (insets) for (a) PdNP and (b) Pd@CisPt.

As shown in Figure 3, a minimal change in the NP size could be detected, from 10 ± 3 nm, for PdNP to 15 ± 2 nm for Pd@CisPt; however a significant change in the contrast of the phase images (insets) was observed. This fact points to a different chemical interaction between the tip and the surface of the NP, before and after the immobilization of CisPt.

In particular, the dark zones for the bare PdNP, likely due to the nanoparticle capping by the soft PVP polymer,^28^ disappear, hidden by a shell visible around the core nanoparticle for the Pd@CisPt. This finding points to the effective binding of cisplatin molecules to the NP’s surface.^29^

As an additional proof of PdNP catalytic properties, the SOD-like assay was carried out in phosphate buffer at pH 7.4. Indeed, Pd nanoparticles can act as artificial antioxidant nanoenzymes (nanozymes) showing similar scavenging activity to that of natural enzymes, including catalase, peroxidase, and SOD, even though such studies are limited to a cell-free environment.^30^ Results indicated an *I*_50_ value, i.e., the concentration that causes 50% inhibition of cytochrome C reduction, of 0.66 nm for PdNP and of 1.84 nM for Pd@CisPt, respectively.

### 3.2 Cellular Experiments Pd@CisPt Nanomaterials

To gain more insight into the performance of Pd@CisPt as a potential theranostic nanomedicine, *in vitro* cellular experiments were carried out in PC-3 prostate cancer cells, to assess the effects of cellular treatments in terms of cytotoxicity, intracellular ROS generation, and cell migration.

**Figure 4:**
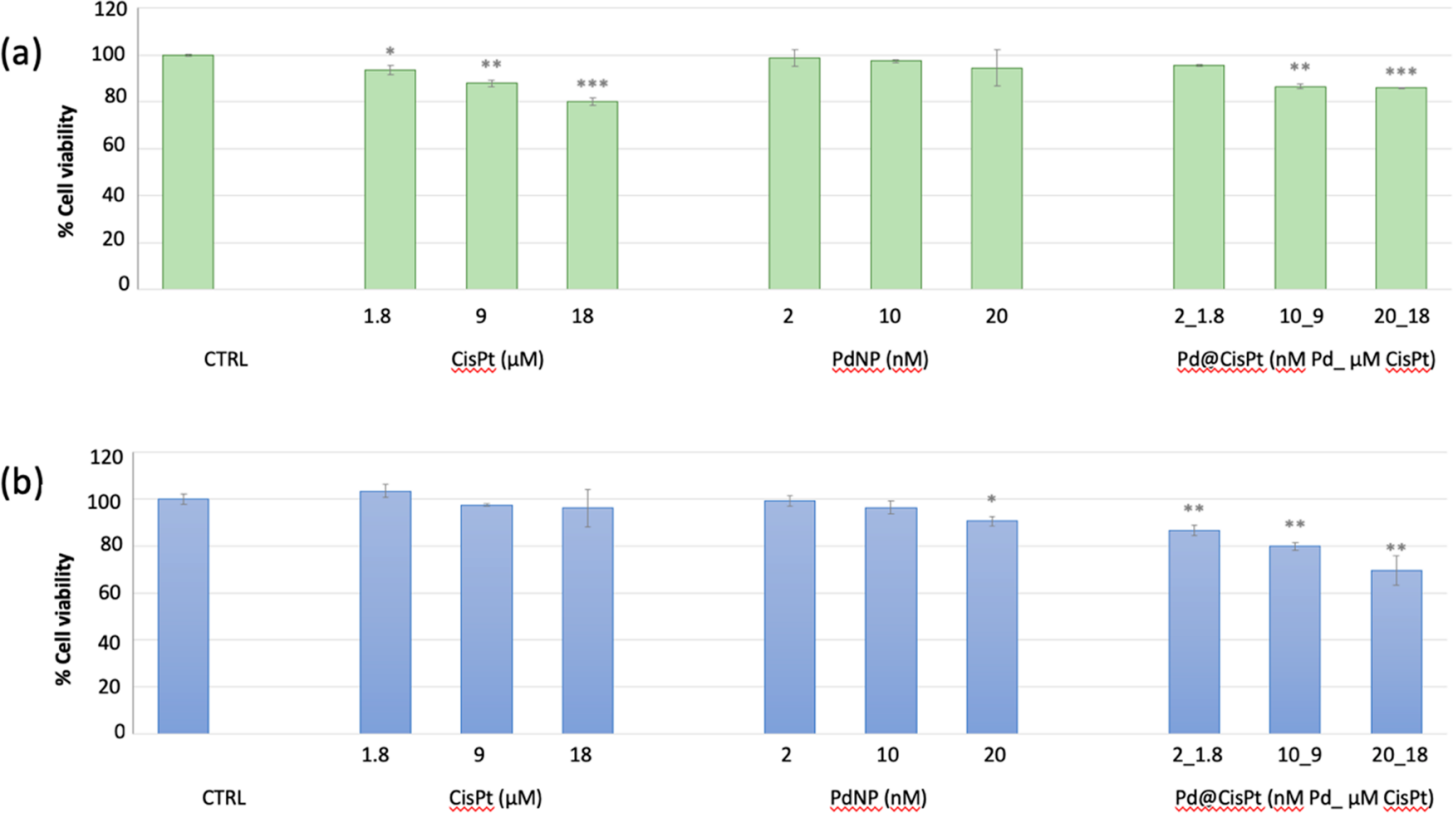
Cell viability measured by (a) nuclear detection (imaging kit Blue/Green) and (b) mitochondrial succinate dehydrogenase activity (MTT assay) in PC-3 cells p.19 treated for 24 h with Pd@CiSt at increasing concentrations. Negative (untreated cells) and positive (i.e., cells treated with cisplatin or PdNP) controls are included. The data are expressed as the average percentage ± SD of three different experiments. Pairwise Student’s *t* test: *p < 0.05; **p < 0.01; ***p < 0.001 vs Ctrl.

In Figure 4 are reported the results of two different cytotoxicity tests, namely, the detection of viable cells by nuclear staining (simultaneous fluorescent staining of dead and total cells using propidium iodide and Hoechst 33342, respectively) or by analysis of mitochondrial succinate dehydrogenase activity (MTT assay). It should be noted that the concentration range for the cellular treatments was kept at low levels for having the possibility to investigate the mechanisms of cytotoxicity (nuclear damage vs ROS-related mitochondrial damage) without devastating effects in terms of cell mortality. Furthermore, under these experimental conditions, we could demonstrate that the hybrid Pd@CisPt is indeed more effective in terms of decreased viability in treated cells (with a dose–response effect) than the free CisPt or the PdNPs alone. Interestingly, cells treated with PdNP did not exhibit a significant decrease of viable cells in the concentration range tested, while a dose–response effect was instead found for both CisPt alone and the Pd@CisPt hybrid. In particular, MTT-measured cell viability pointed to a higher toxic effect than nuclear staining, suggesting a significant perturbation of mitochondrial metabolism.^31^

In fact, mitochondria have been proposed as biomarkers of the response to platinum-based therapies since these organelles play a crucial role in cisplatin sensitivity. In particular, mitochondrial ROS is related to the mitochondrial content, thus a reduction of mitochondrial biogenesis by knock-down of transcription factors is expected to attenuate the cisplatin-induced apoptosis.^6^

In addition to the nuclear DNA damage, CisPt induces production of ROS in target cells, causing damage to cellular structures and altering the normal physiological functions of cells by interacting with various biomolecules, including carbohydrates, nucleic acids, unsaturated fatty acids, and proteins.^6^ Pd nanoparticles can generate free radicals depending on the treated cell line. Specifically, oxidative stress was found for several type of cancer cells, including THP-1 cells, ovarian cancer cells, skin malignant melanoma cells, human leukemia cells, and prostate cancer cells such as PC-3. On the other hand, no significant ROS production was observed for normal cells treated with PdNP, for example, primary bronchial epithelial cells, human eosinophils, and peripheral blood mononuclear cells.^32^

**Figure 5:**
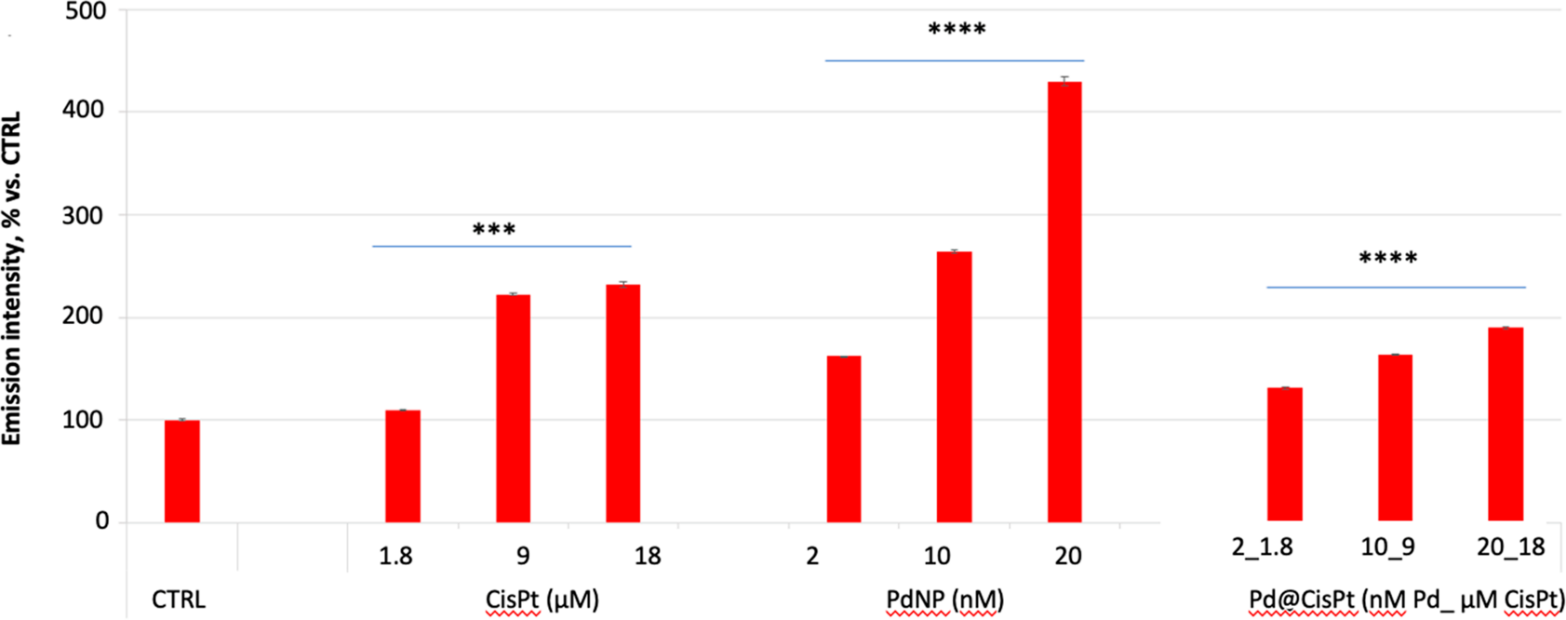
MitoSOX assay’s results for the level of mitochondrial ROS in PC-3. Cells were incubated for 24 h with Pd@CisPt at increasing concentrations. Negative (untreated cells) and positive (i.e., cells treated with cisplatin or PdNP) controls are included. Data are expressed as the MitoSOX ratio with respect to DCF emission intensities (average percentage ± SD of three different experiments). Pairwise Student’s *t* test: ***p < 0.001; ****p < 0.0001 vs Ctrl.

To examine the relevance of ROS production in cisplatin-induced cell death compared to the hybrid Pd@CisPt and the bare PdNP, cells were stained with MitoSOX reactive dye. Results indicated (*i*) an increase of the mitochondrial ROS production induced by all of the samples at the tested conditions and (*ii*) a higher increase of ROS for the bare PdNP in comparison with the hybrid Pd@CisPt, which led to a comparable response to that shown for the free CisPt drug (Figure 5).

Nanoparticle internalization and mitochondrial perturbation were confirmed by confocal microscopy analyses, which also evidenced a correlated alteration of intracellular copper upon the cell treatment with Pd@CisPt (Figure S3 in the SI).

**Figure 6:**
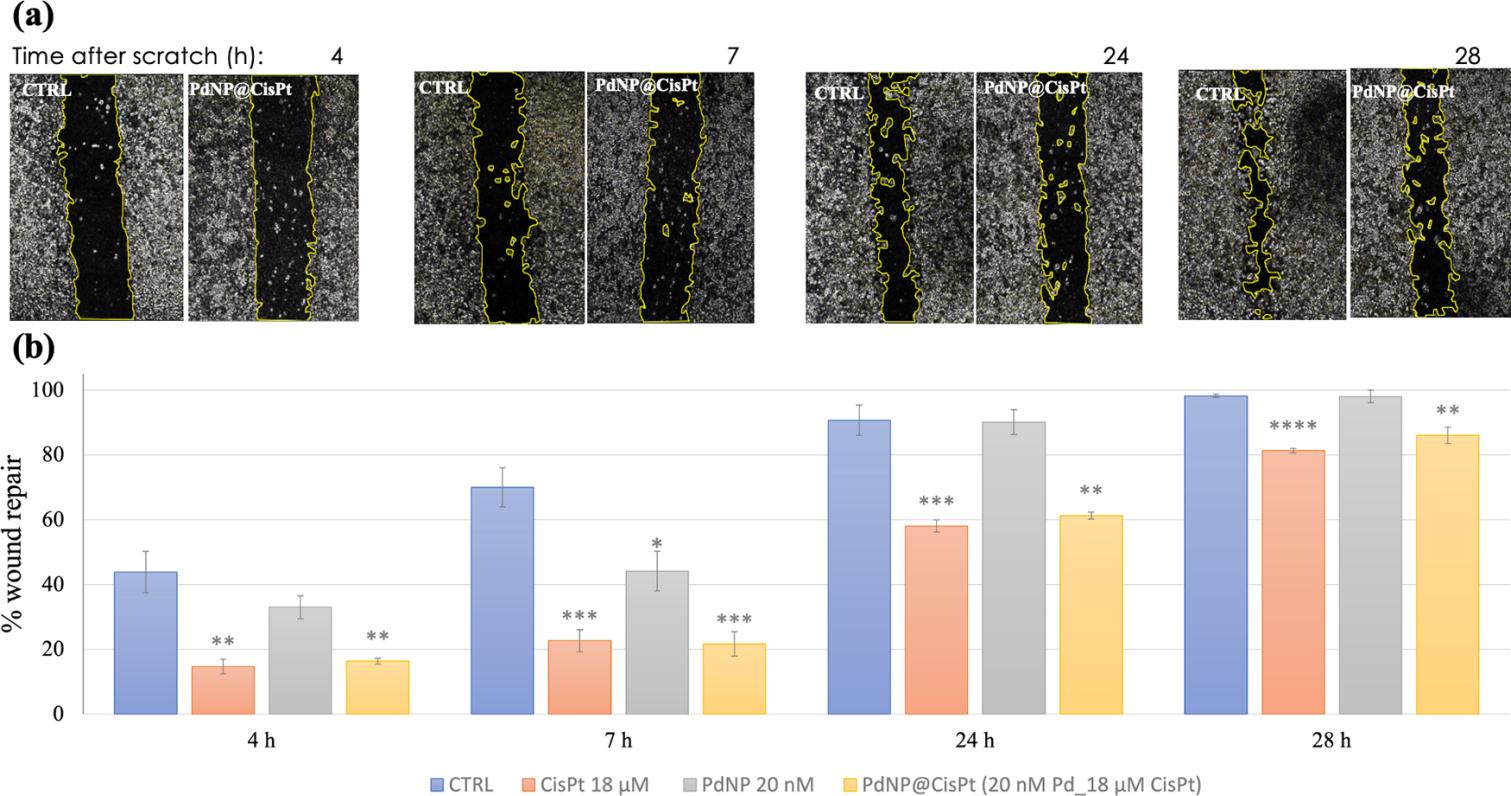
(a) Representative micrographs of PC-3 cells, in the absence (negative control) or in the presence of Pd@CisPt (20 nM nanoparticles, 18 μM drug) hybrid collected at different times (*t* = 4,7,24, 28 h) after scratching (*t* = 0). (b) Quantitative analysis of cell migration (wound edge advancement in percent vs time). Means ± SEM values from three independent experiments. Pairwise Student’s T: *p < 0.05, **p < 0.01, ****p < 0.0001 vs Ctrl.

It is well-known that cisplatin has antitumoral activity and is capable of reducing the migration rate of cancer cells.^33^ Our results of the wound healing test demonstrated the ability of the hybrid to maintain the ability of the drug to inhibit cell migration. In fact, at all times of treatment, Pd@CisPt showed, very similar to CisPt alone, a significant reduction in cell migration rate with respect to the control (Figure 6).

RMS was exploited for a single-cell spectroscopic approach, in combination with the other techniques, to provide further information regarding the effects of Pd@CisPt in the comparison with the positive controls of CisPt and PdNP alone.

The Raman spectra collected in the nuclear region of treated cells are clearly different with respect to those extracted from control ones, as shown by multivariate analysis, evidencing a significant modification in cellular composition induced by the treatments (Figure S4, panels a–d and related discussion in SI). A deeper investigation was carried out by comparing the height of specific Raman peaks, especially related to proteins and nucleic acids (Table S4).

As regards proteins, all the selected peaks were significantly lower in CisPt, PdNP, and Pd@CisPt groups than in Ctrl ones. The decrease in height of the 1030 cm^–1^ peak, assigned to the phenylalanine amino acid, suggests a modification of protein environment induced by the treatments, as it is already reported for other chemotherapy agents.^34^ Similarly, the decreasing trend in height of the peaks at 1280 and 980 cm^–1^, assigned respectively to α-helix and β-sheet structures, suggests that, although all of the treatments induced a loss in protein secondary structures, the action of PdNPs is lower than the one of CisPt. This hypothesis is confirmed by the fact that a minor effect is found for the Pd@CisPt500 treatment, which contains half of the CisPt dose compared to that of the Pd@CisP1000 one. However, the lowest values of all analyzed peaks for Pd@CisP1000 suggest that the combination of PdNP with the highest dose of CisPt is more effective than CisPt alone. Together, these results describe a condition of protein misfolding/unfolding, directly or indirectly due to treatment with cisplatin and/or palladium treatment.^35^

Regarding nucleic acids, the peaks at 1375 and 785 cm^–1^, both assigned to DNA, showed a major decrease in all treated groups, with the latter displaying the lowest height value in the Pd@CisPt1000 group; consistently, the decrease of Hoechst 33342 related peaks (1560 and 1610 cm^–1^) indirectly evidenced a decrease in DNA content,^36^ induced by CisPt and PdNP, with the most effective treatment resulting in the Pd@CisPt1000 group. Furthermore, a change in DNA conformation evidenced by an increase in A-form DNA (810 cm^–1^) and a decrease in B-form DNA (830 cm^–1^) was also detected. In particular, the treatments with Pd@CiSPt500 and Pd@CiSPt1000 showed a particularly marked increase in the misfolded DNA A form, which is also known to occur during drug treatments, especially with intercalating compounds that interrupt base pairing.^37^

To highlight differences in the spectral profiles, pairwise PCA analyses were performed on preprocessed Raman spectra of Ctrl/CisPt, Ctrl/PdNP, and Ctrl/PdNP@Cis groups; results are reported as scores plots (Figure S4, panels e–h), together with the corresponding PC1 loadings (Figure S4, panels i–l). Complete segregation along PC1 was found in all pairwise comparisons, evidencing different spectral characteristics between the control and each treated sample, mainly in nucleic acid and protein components, as confirmed by PC1 loadings.

**Figure 7:**
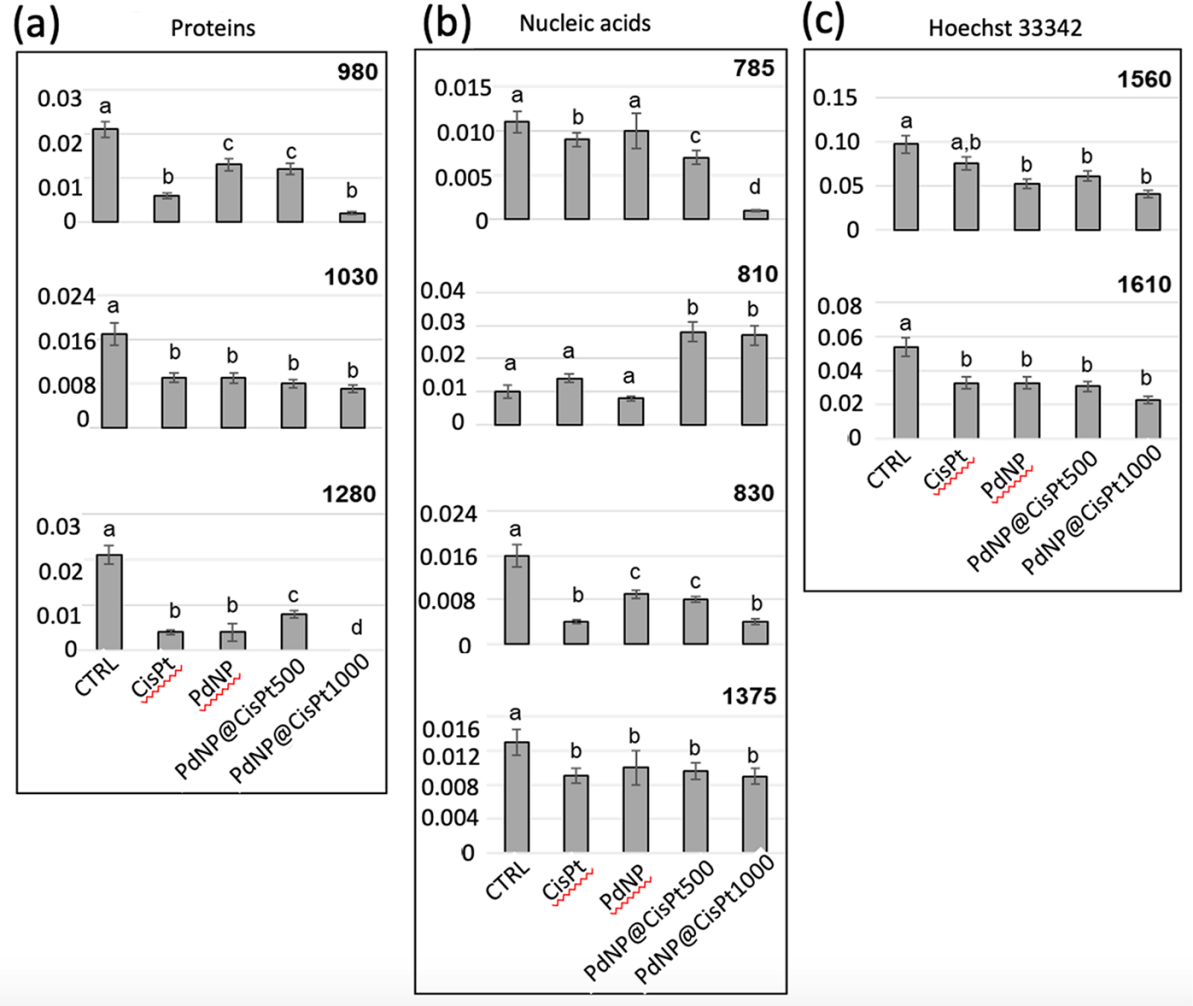
Univariate statistical analysis of peak height values calculated for the control, CisPt, PdNP, Pd@CisPt500, and Pd@CisPt1000 groups and related to (a) proteins (980 cm^–1^, 1030 cm^–1^, and 1280 cm^–1^), (b) nucleic acids (785 cm^–1^, 810 cm^–1^, 830 cm^–1^, and 1375 cm^–1^), and (c) Hoechst 33342 dye (1560 and 1610 cm^–1^). Values are reported as mean ± SD. Significant differences between experimental groups were determined by means of a factorial analysis of variance (one-way ANOVA), followed by Tukey’s multiple comparisons test, by the statistical software Prism6 (Graphpad Software, Inc. USA). Statistical significance was set at *p* < 0.05. Different letters over box charts indicate statistically significant differences among the above-defined experimental groups.

To better understand the biochemical alterations induced by CisPt and PdNP treatments, alone and combined, the height of specific peaks identified by PC1 loadings was calculated and statistically analyzed (Figure 7).

As regards proteins, a significant decrease in 980 cm^–1^, 1030 cm^–1^, and 1280 cm^–1^, respectively representing β-sheet structures, phenylalanine, and α-helix structures, was observed in all of the experimental groups, suggesting some changes in nuclear proteins, induced by the treatments (Figure 7a): with respect to the control, the 1030 cm^–1^ peak showed a significant decrease in all of the treated groups, which shared statistically comparable values; the 1280 cm^–1^ peak displayed a significant decrease in CisPt and PdNP groups with respect to Ctrl, a significant increase in Pd@CisPt500 with respect to CisPt, and the lowest height value in Pd@CisPt1000; the 980 cm^–1^ peak displayed statistically comparable values in PdNP and Pd@CisPt500 groups, and the lowest values in CisPt and Pd@CisPt1000 groups.

As regards nucleic acids, several spectral markers were considered, including the peaks centered at 785 and 1375 cm^–1^ (assigned to DNA), 810 cm^–1^ (assigned to A-form DNA), and 830 cm^–1^ (assigned to B-form DNA; Figure 7b).

In particular, the 1375 cm^–1^ peak displayed a significant decrease with respect to the control in all of the treated groups, which shared statistically comparable values; the 785 cm^–1^ peak displayed a general significant decrease in all of the treated groups, except for the PdNP group, with the lowest value showed by the Pd@CisPt1000 group. The 830 cm^–1^ peak displayed statistically comparable values in the PdNP and Pd@CisPt500 groups and the lowest values in the CisPt and Pd@CisPt1000 groups. The 810 cm^–1^ peak showed a slight but not significant increase in the CisPt group and a significant increase in both the Pd@CisPt groups.

The nuclear DNA content was also indirectly monitored by the analysis of the two peaks related to the Hoechst 33342 dye, specific for DNA (Figure 7c): both of the peaks displayed a significant decrease in all of the treated experimental groups, sharing the trend of the DNA-related peak centered at 1375 cm^–1^.

## 4 Conclusions

In this work, we fabricated monodisperse core–shell spherical palladium nanoparticles by a green synthesis method implemented, for the first time, with PVP as a capping agent, to improve the nanoparticle biocompatibility and stability. CisPt-functionalized PdNP was obtained by physisorption of the drug on the metal surface, and the properties of the hybrid Pd@CisPt nanomaterial were characterized by a multitechnique approach, including UV–vis, ICP-OES, DLS, AFM, and a cell-free xanthine oxidase assay for SOD-like activity. Our results pointed to the very promising properties of the developed nanomaterial for the purpose of a multimodal theranostic platform. In fact, PVP-coated PdNP showed a strong plasmonic band and high photocatalytic activity (almost 20 times the enhancement in solar H_2_ production compared to bare TiO_2_). The hybrid Pd@CisPt construct was proved to be capable of producing mitochondrial ROS and inhibiting the migration of PC-3 prostate cancer cells. Moreover, RMS and PCA analyses ruled out a condition of protein misfolding/unfolding induced by the treatment with Pd@CisPt, as well as a particularly marked increase of the misfolded DNA A-form, which is known to occur also during the drug treatments, especially with intercalating compounds interrupting base pairing.

## Supporting Information Available

Tables S1 and S3 (theoretical calculation of NP concentration and CisPt loading, respectively), Figure S1 (XRD analysis), Figure S2 and Table S2 (photocatalytic H_2_ production analysis), Figure S3 (confocal microscopy analysis), and Figure S4 and Table S4 (Raman spectra analysis) (PDF)
