# Supplementary material for: Pd-Based Hybrid Nanoparticles As Multimodal Theranostic Nanomedicine
Source: ACS Appl Bio Mater. 2023 Jan 18;6(2):483–93. doi: 10.1021/acsabm.2c00759 (PMC9945085; doi:10.1021/acsabm.2c00759)
Supplement: Supplementary file 1 — mt2c00759_si_001.pdf [file mt2c00759_si_001.pdf]

Supplementary Information

Pd-based hybrid nanoparticles as multimodal theranostic nanomedicine

Alberto Bellissima,[▿,†], Lorena M. Cucci[▿,†], Vanessa Sanfilippo[†], Angela De Bonis[‡], Roberto Fiorenza,[§] Salvatore Scirè,[§] Tiziano Marzo,[||] Mirko Severi,[⊥] Diego La Mendola,[||] Valentina Notarstefano,[#] Elisabetta Giorgini,[#] Cristina Satriano[,†]*

[†] *Nano Hybrid BioInterfaces Laboratory (NHBIL), Department of Chemical Sciences, University of Catania, viale Andrea Doria, 6. 95125 Catania, Italy.*

[‡] *Department of Science, University of Basilicata, viale dell'Ateneo Lucano, 10. 85100 Potenza, Italy.*

[§] *Department of Chemical Sciences, University of Catania, viale Andrea Doria, 6. 95125 Catania, Italy.*

[||] *Department of Pharmacy, University of Pisa, via Bonanno Pisano 6, 56126 Pisa, Italy.*

[⊥] *Department of Chemistry ''U. Schiff'', University of Florence, via della Lastruccia 3, 50019 Sesto Fiorentino, Italy.*

[#] *Department of Life and Environmental Sciences, Polytechnic University of Marche, Via Brecce Bianche, 60131 Ancona, Italy.*

** Phone: +39 0957385136. E-mail: cristina-satriano@unict.it*

[▿] *These authors contributed equally.*

Table S1. UV-visible analysis for as prepared, pellet 1 and pellet 2 of PdNP samples. Experimental values of wavelength (λ_{max}, in nm) at the maximum of plasmon peak absorbance (A_{max}) and calculated values of molar extinction coefficient (ε, in $cm^{-1}M^{-1}$), optical diameter (d_{opt}, in nm) and concentration (in nM and NP/mL).

Sample	λ_{max} (nm)	A_{max}	ε ($cm^{-1}M^{-1}$)	d_{opt} (nm)	mol/L $\cdot 10^{-9}$	NP/mL
PdNP, as prepared	273	0.188	$2.0x10^6$	4	35	$1.43x10^{11}$
PdNP, pellet 1	273	0.796	$2.3x10^6$	4	111	$3.85x10^{11}$
PdNP, pellet 2	274	0.704	$3.1x10^6$	5	91	$1.39x10^{11}$

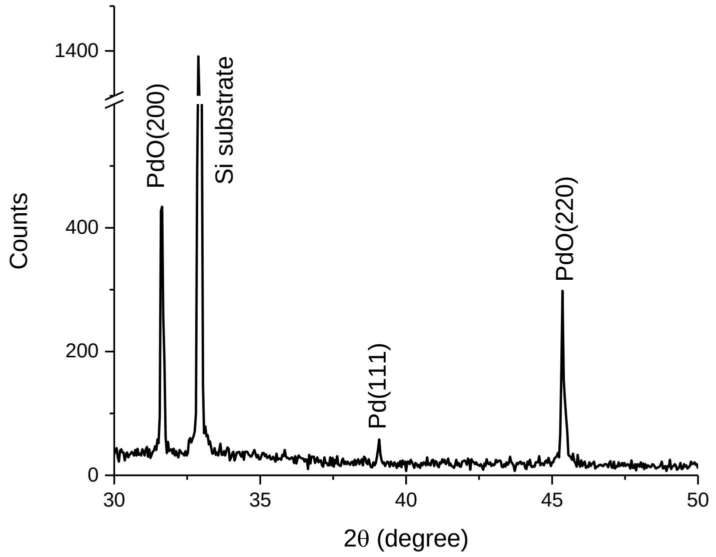

Figure S1. XRD spectrum of PdNP. The signal at 32.9° is referred to silicon substrate.

The diffraction signals at 31.6° and 45.4° and 39.1° can be referred to (200) and (220) crystal planes of cubic PdO (reference code: 00-046-1211) and to (111) crystal planes of cubic Pd (reference code: 01-087-0637), respectively.

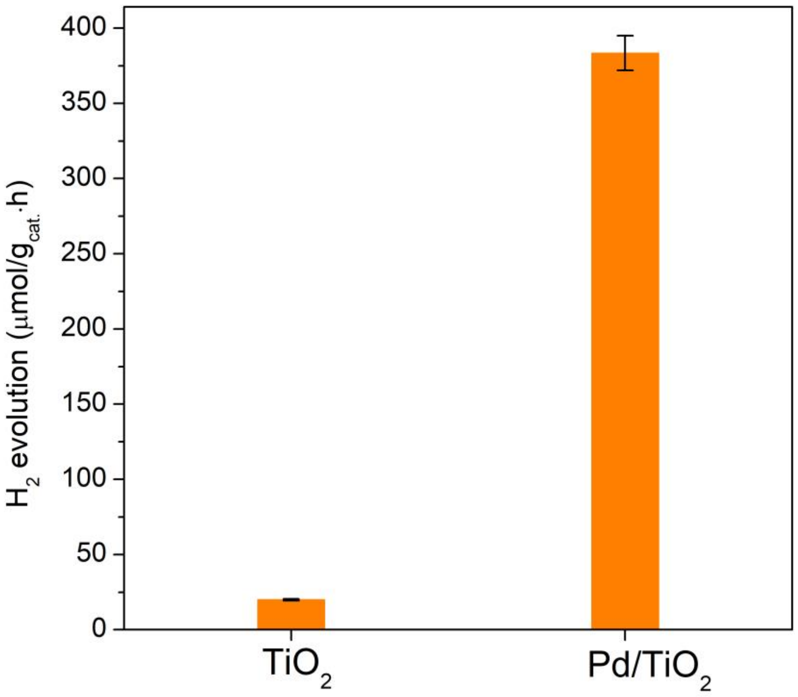

Figure S2. Solar photocatalytic H_2 production of the tested samples expressed in 10^{-6}·mol and normalized for grams of catalyst and hours of irradiation.

Table S2. Literature comparison for the solar photocatalytic H_2 production over some Pd/TiO_2-based samples with our samples (in red).

Sample	Pd content [wt %]	Experimental conditions	Irradiation source	H_2 evolution [μmol/ $g_{cat.}$·h]	[ref]
Pd/TiO_2	0.4	50 mL DMF, 0.5 mL water, 0.5 mL benzylamine	200 mW/cm^2 Xe lamp	400	[1]
Pd@TiO_2/$TiOF_2$	1	60 mL of aqueous solution containing 0.35 MNa_2SO_3, 0.25 M Na_2S	300 W, 50 mW/cm^2, Xe lamp	213	[2]
Pd@TiO_2@Au	0.6	20 mL water, 4 mL ethanol	300 W, 250 mW/cm^2, Xe lamp	272.3	[3]
Au-Pd/TiO_2	0.4	28.5 water, 1.5 mL glycerol	UV flux (320–400 nm) ~12 mW/cm^2, visible	400	[4]

			flux (400–700 nm) ~97 mW/cm^2 Xe lamp		
Pd/TiO_2	0.1	40 mL water, 10 mL glycerol	300 W, 10.7 mW/cm^2 solar lamp	383.5	This work

The concentration of PdNP/mL was calculated considering a face-centered cubic Pd structure[5] and using 0.389 as Pd lattice unit[6]; the number of CisPt molecules for nanoparticle was found dividing Avogadro's number by the PdNP/mL concentration (**Table S3**).

Table S3. Calculated concentrations (in mol/L and NP/mL) of PdNP and number of cisplatin molecules per NP used for the functionalization protocol.

**Sample**	**[PdNP]mol/L**	**[PdNP]/mL**	**[CisPt] mol/L**	**CisPt molecules/NP**
Pd@CisPt	$1.58x10^{-7}$	$3.3x10^{11}$	$9x10^{-4}$	$1.8x10^{12}$

The internalization of PdNP, cisplatin and PdNP@CisPt as well as their interaction with intracellular organelles was studied with confocal laser scanning microscope (LSM) by using the mitochondrial intracellular staining (Mitotracker deep red) and copper (CS1 probe).

As shown in the **Figure S3** PdNP enters the cells, causing an increase in both Mitotracker deep red (Fig.S3c) and CS1 (Fig.S3b) intensities.

PdNP generally localize in different sites which depend on the cells, for instance in *Desulfovibrio desulfuricans* cells they locate at the level of the periplasm, in *Escherichia coli* were observed within the intracellular matrices and membranes, in the HT1080 fibrosarcoma cell line PdNP accumulates in phagocytes while in human peripheral blood

mononuclear cells (PBMCs) within or adjacent to cytoplasmic vesicles.[7, 8] In our case, we noticed particles aggregates spread around the cell cytoplasm and specially located at the cell membrane. Pd ions dissolved from nanoparticles tend to accumulate in mitochondria interfering with the oxidative phosphorylation, thus causing a poor production of ATP and an excessive production of ROS.[7] Such an effect causes an increase of the Mitochondrial activity and so of the organelles stain intensity. The higher CS1 intensity may be explained by the fact that elevated expression of Cu chelators, like glutathione (GSH), upregulates hCtr1 expression. In fact, elevated expression of GSH could be explained by the fact that GSH is an important ROS sensor/regulator, so the oxidative stress-induced mechanism during drug and pro-oxidants treatment could cause the GSH overexpression.[9] Concerning the treatment with the free cisplatin, it enters the cells, leading to a significant increase of the Mitotracker deep red and CS1 intensities, respect to the untreated cells as well as the PdNP. This could be explained because of the main target of cisplatin in cancer cells are mitochondria, in fact it tends to accumulate in them, binding DNA and blocking ATP synthesis.[10] Moreover, cellular uptake of cisplatin appears to be related with copper transport, indeed the most important Cu influx transporter in the cell, Ctr1, seems to be able of transporting cisplatin through methionine rich motifs in the N-terminal extracellular domain, which are fundamental for Cu transport too. Moreover, the metallochaperone Atox1 also seem to be involved, in fact the loss of Atox1 reduces cisplatin influx.[11] Interestingly, the hybrid PdNP@CisPt, compared to bare PdNP and the untreated cells, showed a higher signal of both Mitotracker deep red and CS1 intensity, with a similar behavior to that found for the free cisplatin.

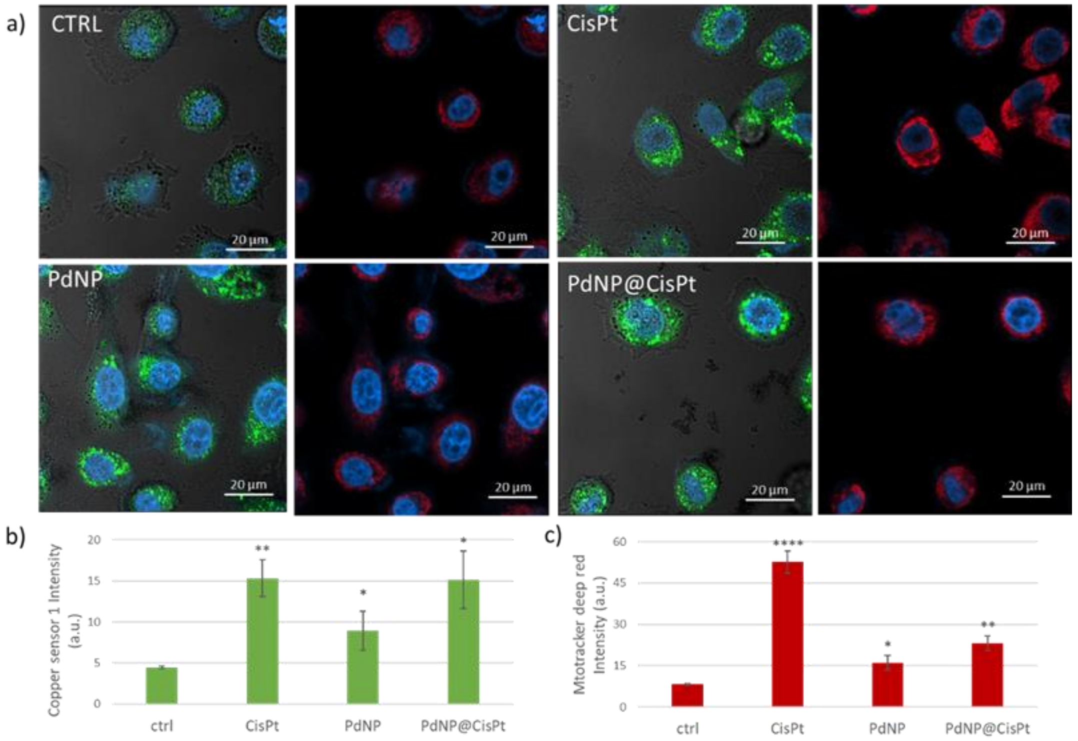

Figure S3: a) LSM fluorescent micrographs of prostate (PC-3) tumour cells: in blue, the nuclear staining with Hoechst ($\lambda_{ex/em}$ = 405/425–450 nm); in green, the CS1 intracellular copper probe ($\lambda_{ex/em}$ = 543/550-600 nm); in red, the MitoTracker deep red ($\lambda_{ex/em}$ = 633/650-700 nm). Cellular treatments were carried out for 90 min with: the negative control of untreated cells, cisplatin, PdNP and PdNP@CisPt. **b)** The graph shows the CS1 intensity in treated and untreated cells, with a higher intensity in treated cells compared to control. **c)** The graph shows the intensity of the Mitotracker deep red in treated and untreated cells, with a higher activity in the treated cells compared to control, particularly for cisplatin and the hybrid PdNP@CisPt. Statistical analysis was performed by pairwise Student's T-test. (*) $p < 0.05$, (**) $p < 0.01$, (***) $p < 0.001$, (****) $p < 0.0001$ vs. CTRL. Scale bar = 20 µm.

The average Raman spectra in the 600-1800 cm^{-1} spectral range are showed in **Figure S4** (panels a-d). Pairwise PCA analyses were performed on pre-processed Raman spectra of Ctrl/CisPt, Ctrl/PdNP and Ctrl/PdNP@Cis groups; results are reported as scores plots (panels e-h), together with the corresponding PC1 loadings (panels i-l).

The PC1 loading of the Ctrl/CisPt comparison (explained variance 44.6%, panel i) displayed the following discriminant spectral features: 785 cm^{-1}, 1095 cm^{-1}, and 1375 cm^{-1} (assigned to DNA); 915 cm^{-1} (assigned to RNA); 1000 cm^{-1}, 1160 cm^{-1}, and 1280 cm^{-1} (assigned to proteins); 1225 cm^{-1} (assigned to proteins and/or nucleic acids); 1450 cm^{-1} (assigned to lipids), and 1560 cm^{-1} and 1610 cm^{-1} (assigned to the Hoechst 33342 dye).

As regards the PC1 loading of the Ctrl/PdNP comparison (explained variance 65.1%, panel j), the following discriminant spectral features were found: 680 cm^{-1}, 1095 cm^{-1}, and 1375 cm^{-1} (assigned to DNA); 810 cm^{-1} (assigned to A-form DNA); 870 cm^{-1}, 980 cm^{-1}, 1000 cm^{-1}, 1208 cm^{-1}, and 1280 cm^{-1} (assigned to proteins); 915 cm^{-1} and 1050 cm^{-1} (assigned to RNA); 1170 cm^{-1} and 1450 cm^{-1} (assigned to lipids), and 1560 cm^{-1} and 1610 cm^{-1} (assigned to the Hoechst 33342 dye) The PC1 loading of the Ctrl/Pd@CisPt comparison (explained variance 53.6% for 1 nM Pd@CisPt500 and explained variance 60.7% for 2 nM Pd@CisPt1000, panels k-l) displayed the following discriminant spectral features: 720 cm^{-1}, 785 cm^{-1} (assigned to DNA); 810 cm^{-1} (assigned to A-form DNA); 830 cm^{-1} (assigned to B-form DNA); 870 cm^{-1}, 980 cm^{-1}, 1030 cm^{-1}, and 1280 cm^{-1} (assigned to proteins); 915 cm^{-1} and 1050 cm^{-1} (assigned to RNA); 1170 cm^{-1} and 1450 cm^{-1} (assigned to lipids), and 1560 cm^{-1} and 1610 cm^{-1} (assigned to the Hoechst 33342 dye).

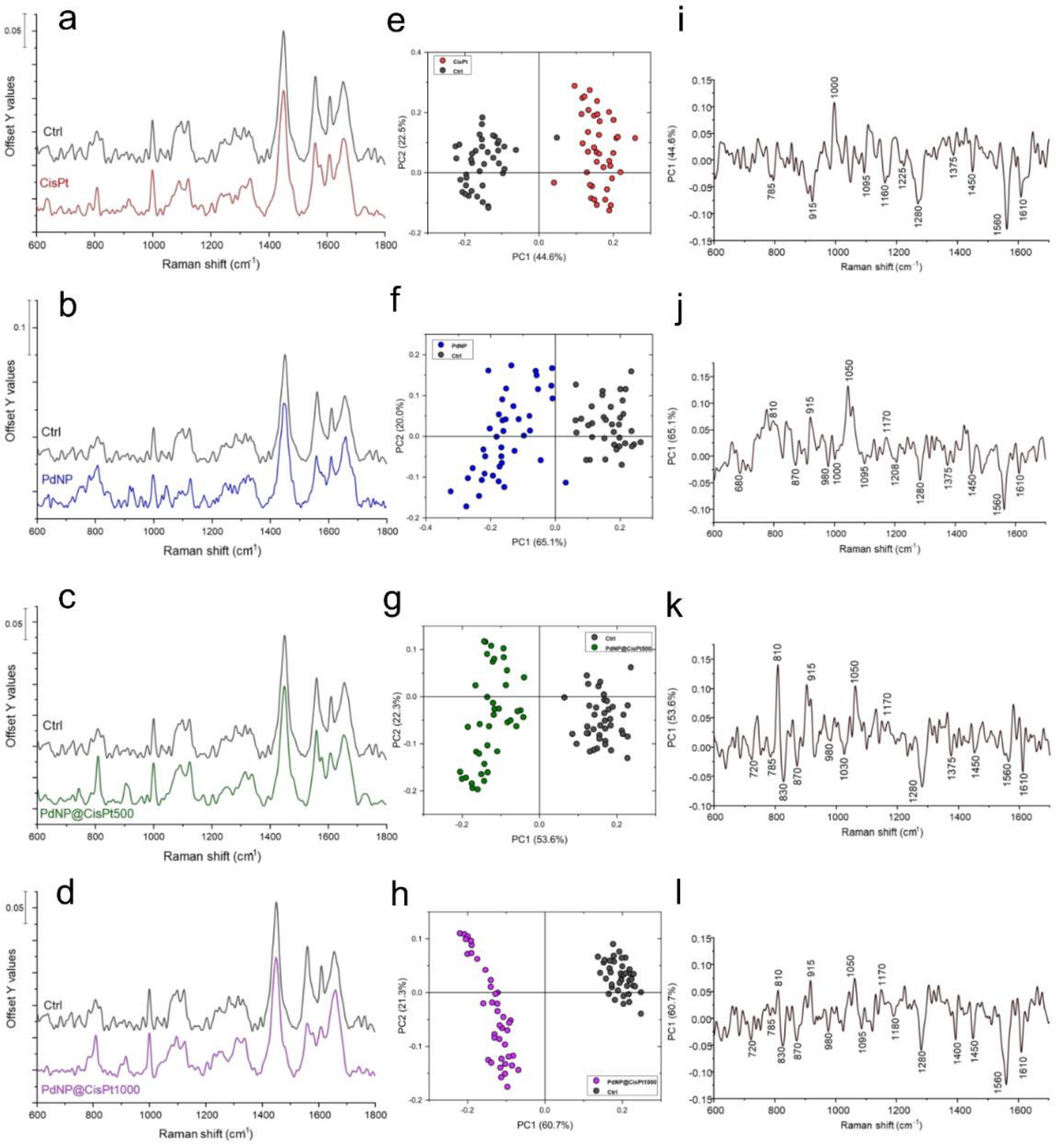

Figure S4. Comparison of average Raman spectra in the 600-1800 cm^{-1} range of: (a) Ctrl and CisPt, (b) Ctrl and PdNP, (c) Ctrl and Pd@CisPt 500, and (d) Ctrl and Pd@CisPt1000. For a better viewing, spectra are off set along y-axis. PCA scores plots (e-h) and PC1 loading spectra (i-l) of: (e, i) Ctrl/CisPt; (f, j) Ctrl/PdNP; (g, k) Ctrl/Pd@CisPt500, and (h, l) Ctrl/Pd@CisPt1000, cellular populations.

The Raman shift of the most relevant peaks, together with the vibrational mode, and the biochemical assignment are reported in **Table S4**.

Table S4. Center position (Raman shifts, cm^{-1}), vibrational mode and biochemical assignment of the peaks identified in average and average ± S.D. absorbance spectra of Ctrl, CisPt, PdNP, Pd@CisPt500, and Pd@CisPt1000 in the 600-1800 cm^{-1} spectral region.

**Raman shift (cm^{-1})**	**Vibrational mode and biochemical assignment**	**Reference**
~680	Ring breathing modes in the DNA bases	[12]
~720	Ring breathing modes of adenine DNA bases	[12, 13]
~785	O-P-O stretching of DNA backbone	[14]
~810	O-P-O stretching of A-form DNA	[15]
~830	O-P-O asymmetric stretching of B-form DNA	[16-18]
~870	C-C stretching of proline	[12, 19]
~915	Ribose vibrations in RNA	[12, 13]
~980	C-C stretching vibration of protein β-sheet structures	[20, 21]
~1000	Symmetric stretching breathing vibration of phenylalanine	[16, 20]
~1030	C-H phenylalanine vibrations	[18, 22]
~1050	RNA P–O stretching, sugar phosphate –C–O– stretching	[18]
~1095	Symmetric PO_2^- stretching vibration of nucleic acids	[12, 17]
~1160	C-C/C-N stretching of proteins	[12, 16, 20, 23]
~1170	C=C stretching and COH bending in lipids	[12]
~1180	Cytosine and guanine vibrations in nucleic acids	[12, 19, 24]
~1208	$C-C_6H_5$ stretching of tryptophan and phenylalanine	[12]
~1225	Amide III, mainly due to β-sheet structures and/or PO_2^- stretching vibration of nucleic acids	[12, 16, 25]
~1280	Amide III, mainly due to helix structures	[12, 16, 20, 23]
~1375	Ring breathing modes of thymine DNA bases	[12, 14, 15-17]
~1400	C=O stretching of aspartic and glutamic acids	[12, 26]
~1450	CH_2 bending modes of lipid chains; bending vibrations of CH_2 of glycerol monooleate	[12, 16]

REFERENCES

(1) Wang, T.; Tao, X.; Li, X.; Zhang, K.; Liu, S.; Li, B. Synergistic Pd Single Atoms, Clusters, and Oxygen Vacancies on TiO_2 for Photocatalytic Hydrogen Evolution Coupled with Selective Organic Oxidation. *Small* **2020**, *17* (2). DOI: 10.1002/smll.202006255.

(2) Zhao, X.; Wei, G.; Liu, J.; Wang, Z.; An, C.; Zhang, J. Synthesis of heterostructured Pd@TiO 2 /TiOF 2 nanohybrids with enhanced photocatalytic performance. *Materials Research Bulletin* **2016**, *80*, 337-343. DOI: 10.1016/j.materresbull.2016.04.018.

(3) She, P.; Qin, J.-s.; Rao, H.; Guan, B.; Yu, J. Spatially separated bimetallic cocatalysts on hollow-structured TiO2 for photocatalytic hydrogen generation. *Materials Chemistry Frontiers* **2020**, *4* (6), 1671-1678. DOI: 10.1039/d0qm00042f.

(4) Khan, M. A.; Sinatra, L.; Oufi, M.; Bakr, O. M.; Idriss, H. Evidence of Plasmonic Induced Photocatalytic Hydrogen Production on Pd/TiO2 Upon Deposition on Thin Films of Gold. *Catalysis Letters* **2017**, *147* (4), 811-820. DOI: 10.1007/s10562-017-1998-4.

(5) Uddin, J. Terahertz multispectral imaging for the analysis of gold nanoparticles' size and the number of unit cells in comparison with other techniques. *International Journal of Biosensors & Bioelectronics* **2018**, *4* (3). DOI: 10.15406/ijbsbe.2018.04.00118.

(6) Sreedhala, S.; Sudheeshkumar, V.; Vinod, C. P. Structure sensitive chemical reactivity by palladium concave nanocubes and nanoflowers synthesised by a seed mediated procedure in aqueous medium. *Nanoscale* **2014**, *6* (13). DOI: 10.1039/c4nr01283f.

(7) Petrarca, C.; Clemente, E.; Di Giampaolo, L.; Mariani-Costantini, R.; Leopold, K.; Schindl, R.; Lotti, L. V.; Mangifesta, R.; Sabbioni, E.; Niu, Q.; et al. Palladium Nanoparticles Induce Disturbances in Cell Cycle Entry and Progression of Peripheral Blood Mononuclear Cells: Paramount Role of Ions. *Journal of Immunology Research* **2014**, *2014*, 1-8. DOI: 10.1155/2014/295092.

(8) Gomez-Bolivar, J.; Mikheenko, I. P.; Macaskie, L. E.; Merroun, M. L. Characterization of Palladium Nanoparticles Produced by Healthy and Microwave-Injured Cells of Desulfovibrio desulfuricans and Escherichia coli. *Nanomaterials* **2019**, *9* (6). DOI: 10.3390/nano9060857. Miller, M. A.; Askevold, B.; Mikula, H.; Kohler, R. H.; Pirovich, D.; Weissleder, R. Nano-palladium is a cellular catalyst for in vivo chemistry. *Nature Communications* **2017**, *8* (1). DOI: 10.1038/ncomms15906.

(9) Kuo, M. T.; Fu, S.; Savaraj, N.; Chen, H. H. W. Role of the Human High-Affinity Copper Transporter in Copper Homeostasis Regulation and Cisplatin Sensitivity in Cancer Chemotherapy: Figure 1. *Cancer Research* **2012**, *72* (18), 4616-4621. DOI: 10.1158/0008-5472.Can-12-0888.

(10) Zhang, Y.; Maurizi, M. R. Mitochondrial ClpP activity is required for cisplatin resistance in human cells. *Biochimica et Biophysica Acta (BBA) - Molecular Basis of Disease* **2016**, *1862* (2), 252-264. DOI: 10.1016/j.bbadis.2015.12.005.

(11) Arnesano, F.; Banci, L.; Bertini, I.; Felli, I. C.; Losacco, M.; Natile, G. Probing the Interaction of Cisplatin with the Human Copper Chaperone Atox1 by Solution and In-Cell NMR Spectroscopy. *Journal of the American Chemical Society* **2011**, *133* (45), 18361-18369. DOI: 10.1021/ja207346p.

(12) Talari, A. C. S.; Movasaghi, Z.; Rehman, S.; Rehman, I. u. Raman Spectroscopy of Biological Tissues. *Applied Spectroscopy Reviews* **2014**, *50* (1), 46-111. DOI: 10.1080/05704928.2014.923902.

(13) Chan, J. W.; Taylor, D. S.; Zwerdling, T.; Lane, S. M.; Ihara, K.; Huser, T. Micro-Raman Spectroscopy Detects Individual Neoplastic and Normal Hematopoietic Cells. *Biophysical Journal* **2006**, *90* (2), 648-656. DOI: 10.1529/biophysj.105.066761.

(14) Ramos, I. R.; Meade, A. D.; Ibrahim, O.; Byrne, H. J.; McMenamin, M.; McKenna, M.; Malkin, A.; Lyng, F. M. Raman spectroscopy for cytopathology of exfoliated cervical cells. *Faraday Discussions* **2016**, *187*, 187-198. DOI: 10.1039/c5fd00197h. Li, N.; Chen, D.; Xu, Y.; Liu, S.; Zhang, H. Confocal Raman micro-spectroscopy for rapid and label-free detection of maleic acid-induced variations in human sperm. *Biomedical Optics Express* **2014**, *5* (5). DOI: 10.1364/boe.5.001690.

(15) Nawaz, H.; Bonnier, F.; Knief, P.; Howe, O.; Lyng, F. M.; Meade, A. D.; Byrne, H. J. Evaluation of the potential of Raman microspectroscopy for prediction of chemotherapeutic response to cisplatin in lung adenocarcinoma. *The Analyst* **2010**, *135* (12). DOI: 10.1039/c0an00541j.

(16) Notarstefano, V.; Sabbatini, S.; Pro, C.; Belloni, A.; Orilisi, G.; Rubini, C.; Byrne, H. J.; Vaccari, L.; Giorgini, E. Exploiting fourier transform infrared and Raman microspectroscopies on cancer stem cells from oral squamous cells carcinoma: new evidence of acquired cisplatin chemoresistance. *The Analyst* **2020**, *145* (24), 8038-8049. DOI: 10.1039/d0an01623c.

(17) Farhane, Z.; Nawaz, H.; Bonnier, F.; Byrne, H. J. In vitro label-free screening of chemotherapeutic drugs using Raman microspectroscopy: Towards a new paradigm of spectralomics. *Journal of Biophotonics* **2018**, *11* (3). DOI: 10.1002/jbio.201700258.

(18) Farhane, Z.; Bonnier, F.; Byrne, H. J. Monitoring doxorubicin cellular uptake and trafficking using in vitro Raman microspectroscopy: short and long time exposure effects on lung cancer cell lines. *Analytical and Bioanalytical Chemistry* **2016**, *409* (5), 1333-1346. DOI: 10.1007/s00216-016-0065-0.

(19) Stone, N.; Kendall, C.; Smith, J.; Crow, P.; Barr, H. Raman spectroscopy for identification of epithelial cancers. *Faraday Discussions* **2004**, *126*. DOI: 10.1039/b304992b.

(20) Notarstefano, V.; Gioacchini, G.; Byrne, H. J.; Zacà, C.; Sereni, E.; Vaccari, L.; Borini, A.; Carnevali, O.; Giorgini, E. Vibrational characterization of granulosa cells from patients affected by unilateral ovarian endometriosis: New insights from infrared and Raman microspectroscopy. *Spectrochimica Acta Part A: Molecular and Biomolecular Spectroscopy* **2019**, *212*, 206-214. DOI: 10.1016/j.saa.2018.12.054.

(21) Notingher, I.; Green, C.; Dyer, C.; Perkins, E.; Hopkins, N.; Lindsay, C.; Hench, L. L. Discrimination between ricin and sulphur mustard toxicity in vitro using Raman spectroscopy. *Journal of The Royal Society Interface* **2004**, *1* (1), 79-90. DOI: 10.1098/rsif.2004.0008.

(22) Notarstefano, V.; Pisani, M.; Bramucci, M.; Quassinti, L.; Maggi, F.; Vaccari, L.; Parlapiano, M.; Giorgini, E.; Astolfi, P. A vibrational in vitro approach to evaluate the potential of monoolein nanoparticles as isofuranodiene carrier in MDA-MB 231 breast cancer cell line: New insights from Infrared and Raman microspectroscopies. *Spectrochimica Acta Part A: Molecular and Biomolecular Spectroscopy* **2022**, *269*. DOI: 10.1016/j.saa.2021.120735.

(23) Huang, N.; Short, M.; Zhao, J.; Wang, H.; Lui, H.; Korbelik, M.; Zeng, H. Full range characterization of the Raman spectra of organs in a murine model. *Optics Express* **2011**, *19* (23). DOI: 10.1364/oe.19.022892. Rygula, A.; Majzner, K.; Marzec, K. M.; Kaczor, A.; Pilarczyk, M.; Baranska, M. Raman spectroscopy of proteins: a review. *Journal of Raman Spectroscopy* **2013**, *44* (8), 1061-1076. DOI: 10.1002/jrs.4335.

(24) Patel, I. I.; Martin, F. L. Discrimination of zone-specific spectral signatures in normal human prostate using Raman spectroscopy. *The Analyst* **2010**, *135* (12). DOI: 10.1039/c0an00518e.

(25) Yosef, H. K.; Frick, T.; Hammoud, M. K.; Maghnouj, A.; Hahn, S.; Gerwert, K.; El-Mashtoly, S. F. Exploring the efficacy and cellular uptake of sorafenib in colon cancer cells by Raman micro-spectroscopy. *The Analyst* **2018**, *143* (24), 6069-6078. DOI: 10.1039/c8an02029a.

(26) Schulz, H.; Baranska, M. Identification and quantification of valuable plant substances by IR and Raman spectroscopy. *Vibrational Spectroscopy* **2007**, *43* (1), 13-25. DOI: 10.1016/j.vibspec.2006.06.001.
